# ZBED4, a cone and Müller cell protein in human retina, has a different cellular expression in mouse

**Published:** 2011-07-21

**Authors:** Mehrnoosh Saghizadeh, Yekaterina Gribanova, Novrouz B. Akhmedov, Debora B. Farber

**Affiliations:** 1 Jules Stein Eye Institute, UCLA, Los Angeles, CA; 2 Molecular Biology Institute, UCLA, Los Angeles, CA

## Abstract

**Purpose:**

ZBED4, a protein in cones and Müller cells of human retina, may play important functions as a transcriptional activator of genes expressed in those cells or as a co-activator/repressor of their nuclear hormone receptors. To begin investigating these potential roles of ZBED4, we studied the developmental expression and localization of both the *Zbed4* mRNA and protein of mouse retina.

**Methods:**

northern blots showed the presence of *Zbed4* mRNA in retina and other mouse tissues, and western blots showed the nuclear and cytoplasmic expression of Zbed4 at different developmental times. Antibodies against Zbed4 and specific retinal cell markers were used for retinal immunohistochemistry.

**Results:**

*Zbed4* mRNA was present at different levels in all the mouse tissues analyzed. The Zbed4 protein was barely detectable at embryonic day (E)14.5 but was clearly seen at E16 at both retinal outer and vitreal borders and throughout the retina by E18 and postnatal day 0 (P0). Thereafter, Zbed4 expression was more restricted to the inner retina. While ZBED4 is localized in cones and endfeet of Müller cells of human retina, in adult mouse retina Zbed4 is only detected in Müller cell endfeet and processes. The same localization of Zbed4 was observed in rat retina. In early development, Zbed4 is mainly present in the nuclear fraction of the mouse retina, and in adulthood it becomes more enriched in the cytoplasmic fraction.

**Conclusions:**

The patterns of spatial and temporal expression of *Zbed4* in the mouse retina suggest a possible involvement of this protein in retinal morphogenesis and Müller cell function.

## Introduction

Recently we reported the isolation and characterization of a novel protein, ZBED4, which belongs to the BED subclass of zinc finger proteins and is expressed in cone photoreceptors and Müller cells (mainly detected in their endfeet) of human retina [1]. ZBED4 has four BED-type zinc fingers, each formed by 50–60 amino acids, two of which are highly conserved aromatic amino acids located N-terminal to the BED signature Cx_2_Cx_n_Hx_3–5_[H/C] (x_n_ is a variable spacer) [2]. Zinc finger domains are common in transcription factor proteins. They play a variety of essential roles in cell growth, differentiation, and development in accordance with their structural diversity [3,4]. BED fingers have been shown to be present in chromatin-boundary element-binding proteins [2]. In addition to BED fingers, ZBED4 also has an hATC dimerization domain [5] and two nuclear hormone receptor-interacting modules [6,7]. Similar to its human counterpart, the mouse *Zbed4* gene encodes a protein of 1,168 amino acids with a molecular mass of approximately 135 kDa. This protein has 82% amino acid identity with human ZBED4 and is highly conserved across species. The *Zbed4* gene maps to mouse chromosome 15 in a region that is syntenic to human chromosome 22q13.3, where the human *ZBED4* gene maps. In this paper we report the localization and developmental expression of Zbed4 in mouse retina. Interestingly, Zbed4 is not detected in cone cells of the mouse, in contrast with its localization in cones of human retina. Moreover, Zbed4 is abundant throughout the embryonic retina but is present only in Müller cells of the adult mouse retina.

## Methods

### Animal tissues

C57Bl/6J mice were obtained from colonies bred from stock originating at the Jackson Laboratories (Bar Harbor, ME). Mice were reared under dim cyclic light and sacrificed by CO_2_ inhalation at the appropriate postnatal day (P). Wistar rats were obtained from Charles River (Wilmington, MA). Eyes of mice and rats were quickly enucleated after death, and retinas were rapidly dissected and frozen on dry ice or fixed in 4% paraformaldehyde. All experiments were conducted in accordance with the Animal Care and Use Committee of the University of California, Los Angeles and the Association for Research in Vision and Ophthalmology statement for the Use of Animals in Ophthalmic and Vision Research.

### RNA isolation

Total RNA was extracted from mouse retinas using Trizol (Invitrogen, Carlsbad, CA). Poly A^+^ RNA was obtained using the Oligotex mRNA purification kit (Qiagen, Valencia, CA). RNA quality was determined with a bioanalyzer (Agilent model 2010, Agilent Technologies, Palo Alto, CA) and quantification was performed using a NanoDrop spectrophotometer (NanoDrop Technologies, Wilmington, DE) [1]. RNA was stored at –80 °C.

### RT–PCR and cloning

DNA-free total RNA was reverse transcribed and the first-strand cDNA was amplified by PCR using the Advantage 2 polymerase mix (BD Bioscience, Franklin Lakes, NJ), 40 mM Tricine–KOH (pH 9.2), 15 mM CH_3_CO_2_K, 3.5 mM (CH_3_CO_2_)_2_Mg, 3.75 μg/ml BSA (BSA), 0.2 mM deoxyribonucleotide triphosphate (dNTP), and 0.2 μM appropriate PCR primers following the same program profile as described before [1]: 94 °C for 2 min; 35 cycles at 95 °C for 30 s, 60 °C for 30 s and 72 °C for 1 min, followed by 72 °C for 10 min. The resulting PCR products were purified using a PCR gel extraction kit (Qiagen, Valencia, CA), cloned into the PCR II vector using a Topo Cloning kit (Invitrogen, Carslbad, CA), and sequenced in an Applied Biosystems ABI PRISM 3100 Genetic Analyzer (Foster City, CA).

### Northern blot analysis

Two micrograms of polyA^+^ RNA obtained from adult mouse retinal tissue were electrophoresed on a 1.2% denaturing formaldehyde agarose gel and transferred to a Hybond N+ membrane (Amersham Biosciences, Piscataway, NJ) [1]. An amplified mouse retinal *Zbed4* cDNA fragment containing part of the 3′ coding region and of the 3′ UTR (3461–4191 bp) was labeled with ^32^P-dCTP and Klenow enzyme and used to hybridize a multiple mouse tissue (Ambion, Austin, TX) and the retinal northern blots.

### Generation of a mouse Zbed4 antibody

A polyclonal antibody was produced in rabbit against a peptide from mouse Zbed4 containing amino acids 8–25 (ProSci, Poway, CA). After affinity purification, the specificity of the antibody for Zbed4 was determined in a similar way to that described for the human ZBED4 antibody [1].

### Protein extraction and immunoblotting

Nuclear and cytoplasmic protein extracts from mouse retinal tissues at different developmental ages were prepared using NE-PER Nuclear and Cytoplasmic Extraction Reagents (Thermo Scientific, Rockford, IL). Quantification of total protein was performed with the Micro BCA Protein Assay Kit (Thermo Scientific). Protein extracts (50 µg) were separated by sodium dodecyl sulfate PAGE on 7.5% gels (Pierce, Protein Research Products, Thermo Scientific) and transferred to polyvinylidene difluoride membranes. The membranes were blocked for 1 h in TBS-T solution (50 mM Tris-HCl [pH 8.0], 150 mM NaCl, 0.1% Tween-20) containing 3% BSA and incubated 2 h at room temperature with the rabbit primary antibody against mouse Zbed4 at a dilution of 1:1,000, followed by 1 h incubation with secondary anti-rabbit IgG antibodies labeled with alkaline phosphatase (1:5,000 dilution, Vector Laboratories, Burlingame, CA). Western blots were visualized with the alkaline phosphatase chemiluminescent DuoLux kit (Vector Laboratories).

### Immunostaining of retinal sections

After dissection and fixation overnight in 4% paraformaldehyde, mouse and rat retinas were embedded in optical cutting temperature compound (Sakura Finetek USA Inc., Torrance, CA). Frozen sections (8 μm) were cut on a Leica CM1850 cryostat (McBain Instruments, Chatsworth, CA). Immunostaining was performed as described previously [1], incubating the sections for 2 h with primary antibodies and subsequently for 1 h with secondary antibodies. The following primary and secondary antibodies were used: rabbit polyclonal against Zbed4 and secondary Alexa 488 donkey anti-rabbit IgG (green, Invitrogen); goat polyclonal antibodies against Brn3a (Chemicon, Millipore, Temecula, CA) and glutamine synthetase (Santa Cruz Biotechnology, Santa Cruz, CA) and for both, secondary Alexa 568 donkey anti-goat IgG (red, Invitrogen); mouse monoclonal against Pax 6 (Abcam, Cambridge, MA) and secondary Alexa 568 goat anti-mouse IgG (red, Invitrogen); and chicken antibodies against green/red and blue cone opsins (a gift from Dr. Jeremy Nathans, Johns Hopkins University, Baltimore, MD) and secondary Alexa 568 goat anti-chicken IgG (red, Invitrogen). Routine controls, such as the use of pre-immune serum instead of primary antibody or sections incubated without primary antibodies, were included with each experiment. Also, Zbed4 rabbit polyclonal antibody pre-absorbed by the Zbed4 peptide used to generate the antibody was used to immunostain tissues.

## Results

### *Zbed4* mRNA is expressed in mouse retina

**Figure 1:**
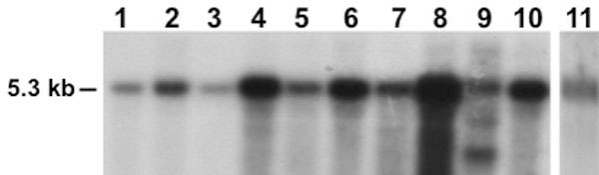
Northern Blots of *Zbed4* mRNA in various mouse tissues. Mouse mRNAs from a multitissue blot and mRNAs from adult mouse retina were probed with a ^32^P-labeled ZBED4 cDNA fragment. A single transcript of approximately 5.3 kb is shown in all tissues except in testis where two smaller bands are also observed. 1, heart; 2, brain; 3, liver; 4, spleen; 5, kidney; 6, embryo, 7, lung; 8, thymus; 9, testis; 10, ovary; 11, retina.

Two mouse *Zbed4* mRNAs are recorded in GenBank: a 5.3-kb mRNA (GenBank BC052174) and a 5.4-kb mRNA (GenBank NM_181412). Alignment of the 5.3-kb mRNA with the *Zbed4* genomic sequence shows that it is derived from two exons, one short noncoding and one long exon that contains part of the 5′ UTR and the complete 3′ UTR. The 5.4-kb mRNA includes an additional 92-bp noncoding exon upstream of the first exon of the 5.3-kb *Zbed4* mRNA. Both mRNAs have the same coding region and 3′ UTR. A 731-bp probe (3461–4191 bp of the 5.3-kb mRNA) encompassing the 3′ coding region and part of the 3′ UTR of the mouse sequence were amplified and used to hybridize a mouse multitissue northern blot (Ambion) along with a membrane containing mouse retinal mRNA. A major transcript approximately 5.3–5.4-kb long (consistent with the length of mouse *Zbed4* mRNAs) was detected in all tissues examined: heart, brain, liver, spleen, embryo, lung, thymus, testis, ovary, and retina (Figure 1). Highest expression was observed in thymus, followed by spleen, the whole embryo, and ovary (Figure 1, lanes 8, 4, 6, and 10, respectively). Moderate levels were detected in brain, kidney, lung, and testis (Figure 1, lanes 2, 5, 7, and 9, respectively), the latter having in addition two other smaller transcripts; and lesser amounts were seen in heart and liver (Figure 1, lanes 1 and 3, respectively). Since the Ambion multitissue blot had 2 μg of mRNA per lane, the same amount of mouse retina mRNA was loaded in our northern blot. Comparison of the hybridization results indicated that *Zbed4* mRNA is not as abundant in retina as in thymus but it may be present in levels similar to those of brain and testis.

### Western blot analysis of Zbed4 expression during mouse retinal postnatal development

**Figure 2:**
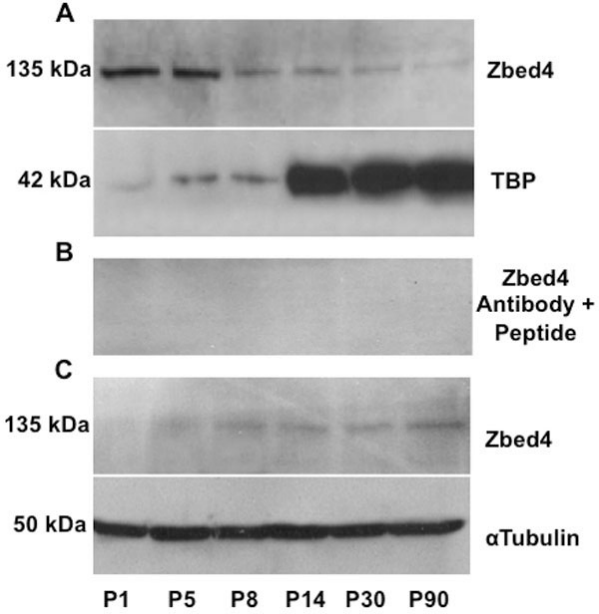
Nuclear and cytoplasmic expression of Zbed4 during mouse retinal development is shown in western blots of retinal protein extracts of mice ranging in age from postnatal day 1 (P1) to P90, using the Zbed4 antibody. **A**: Nuclear Zbed4 expression decreased from birth to adulthood. Also shown is the expression pattern of TBP at all the time points studied, which demonstrates that TBP cannot be used as a loading control for nuclear Zbed4. **B**: To confirm the specificity of the Zbed4 antibody, a competition reaction was performed. The antibody was incubated overnight with a 3,000 molar excess of the peptide used to generate it. A duplicate of the western blot shown in A was incubated with the pre-absorbed Zbed4 antibody. No Zbed4 bands are detected in the retinal nuclear extracts from mice of different developmental stages. **C**: Cytoplasmic Zbed4 expression increases from P1 to P90. α-Tubulin was used as a loading control.

To analyze the nuclear and cytoplasmic distribution of Zbed4 in mouse retinas at different times of postnatal development, immunoblotting of the separated proteins from nuclear and cytoplasmic retinal extracts was performed at several ages between P1 and P90, using the highly specific Zbed4 antibody. In agreement with its expected size, a band of 135 kDa corresponding to Zbed4 was detected both in nuclear (Figure 2A) and cytoplasmic (Figure 2C) retinal extracts. To corroborate the specificity of the Zbed4 antibody, a duplicate blot of that in Figure 2A was incubated with the Zbed4 antibody that had been pre-absorbed by the peptide used to generate it. Figure 2B shows no Zbed4 band in any lane of the western blot, attesting to the Zbed4 antibody specificity.

The TATA-box-binding protein (TBP) is commonly used to normalize the amount of nuclear protein loaded in each lane of a gel [8,9]. However, we found that in mouse retinal nuclear extracts, TBP is expressed at one level up to P14 and at a different level thereafter (Figure 2A). Therefore, we could not use TBP as a normalizer for nuclear retinal proteins during development. Since the cytoplasmic retinal extracts showed a consistent level of α-tubulin (their normalizer) in all lanes of the gel, we assume that the nuclear extracts were also evenly loaded. This being the case, we infer from Figure 2A that Zbed4 expression is mainly nuclear in early development, with levels decreasing with age, while Figure 2C indicates that the cytoplasmic Zbed4, which is barely detected at P1, increases from early life and reaches the highest levels in the adult retina.

### Immunohistochemical localization of Zbed4 during retinal development

**Figure 3:**
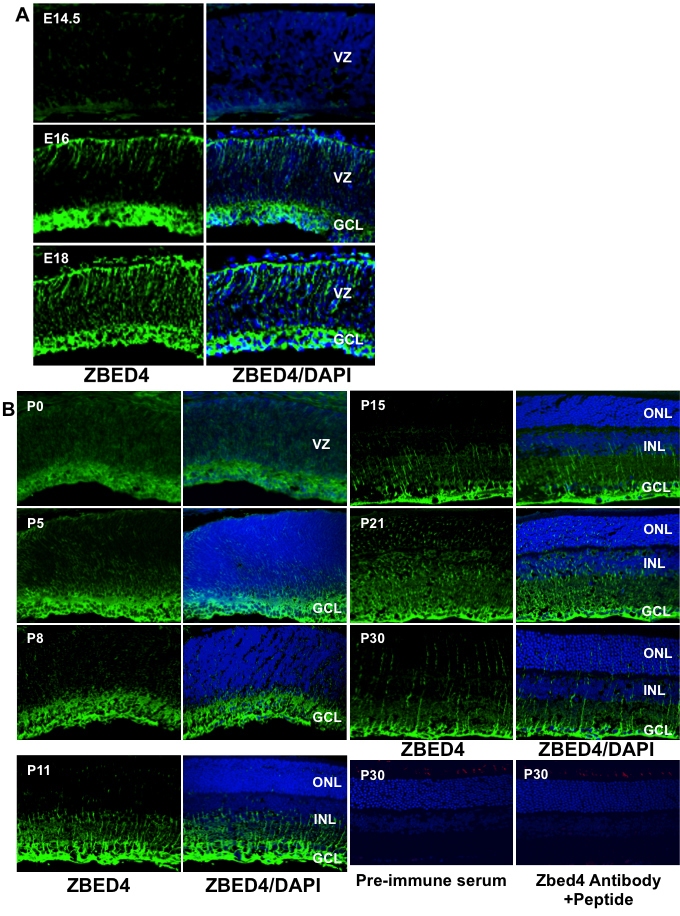
Developmental pattern of Zbed4 immunoreactivity in mouse retina. From embryonic life through adulthood, Zbed4 is observed only in Müller cells of the mouse retina. Retinal sections from embryonic day (E)14.5, E16, E18 (**A**), P0, P5, P8, P11, P15, P21, and P30 (**B**) animals were incubated with rabbit polyclonal Zbed4 antibody followed by fluorescein isothiocyanate-conjugated goat anti-rabbit antibodies and 4',6-diamidino-2-phenylindole (DAPI) to visualize nuclei. Merged images of Zbed4 antibody and DAPI stainings are shown in the right panel of each set. Acronyms: VZ, ventricular zone; GCL, ganglion cell layer; ONL, outer nuclear layer; INL, inner nuclear layer. Magnification is 400×. Mouse retinal sections of all time points were also reacted with pre-immune serum followed by secondary antibody as a control for nonspecific binding. Only one of these control sections is shown for P30. This section was also incubated with a chicken antibody against green/red cone opsin followed by reaction with Alexa 568 goat anti-chicken IgG. In addition, retinal sections from mice of all ages were incubated with Zbed4 antibody that was pre-absorbed by the peptide used to generate it, and with antibody against green opsin and DAPI. Although the green cones can be identified by their red color, no staining for Zbed4 was observed, attesting to the specificity of the Zbed4 antibody. Only one of these control sections is shown for P30.

To investigate the expression of Zbed4 at different times during embryonic and postnatal development, immunostaining was performed on mouse retinal sections at embryonic day (E)14.5, E16, E18, P0 (with the first 24 h after birth defined as P0), P5, P8, P11, P15, P21, and P30, using the Zbed4 antibody. As seen in Figure 3A, Zbed4 protein expression is barely detectable at E14.5 but it is seen at both the outermost (choroidal) and innermost (vitreal) borders of the retina at E16 and it extends throughout the retina from E18 to P0 (Figure 3A,B). Between P5 and P8, Zbed4 expression appears to become more localized to the innermost retinal layer, and the same pattern remains thereafter (Figure 3B). At P11, Zbed4 stains Müller cell processes that are reaching the inner nuclear layer, and by P15 they have extended to the outer nuclear layer. From P21 onward, Zbed4 is expressed in Müller cell processes across the outer nuclear layer and, as at P15, is also strongly localized to their endfeet. Control sections incubated with rabbit pre-immune serum and with Zbed4 antibody that had been pre-absorbed with a 3,000 molar excess of the Zbed4 peptide used to generate it showed no positive signal.

### Zbed4 protein does not co-localize with green or blue opsins in mouse retina

**Figure 4:**
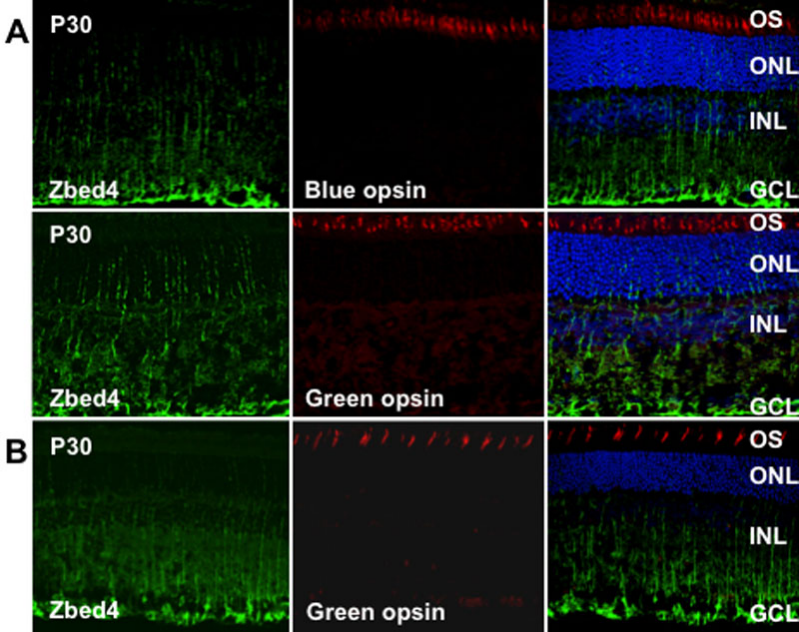
Zbed4 does not co-localize with green and blue opsins in adult mouse and rat retinas. Confocal microscopy of mouse (**A**) and rat (**B**) retinal sections were double labeled with antibodies against Zbed4 and green/red or blue opsin, followed by Alexa 488 goat anti-rabbit IgG (green) and Alexa 568 goat anti-chicken IgG (red). The sections were then stained with DAPI (blue). Cone outer segments are stained red by opsin antibodies. Magnification is 400×. Acronyms: OS, outer segments; ONL, outer nuclear layer; INL, inner nuclear layer; GCL, ganglion cell layer.

We previously showed that ZBED4 is mostly present in the nucleus and inner segment of cones and is detected in the endfeet of Müller cells of human retina [1]. In the present study we performed immunohistochemistry studies on frozen retinal sections of adult mouse, double labeling the sections with the polyclonal antibody against mouse Zbed4 and antibodies against green/red or blue cone opsins. Contrary to our expectation, the majority of Zbed4 staining was found in the innermost retinal layer, at the endfeet of Müller cells, and in their processes (Figure 4A). No co-localization was observed with either of the two cone opsins. We also investigated if similar results were observed in rat retina and found that, at P30, rat retina had the same Zbed4 localization as the P30 mouse. Furthermore, rat Zbed4 did not co-localize with green opsin (Figure 4B).

### Co-localization of Zbed4 with Müller cell markers in developing mouse retina

**Figure 5:**
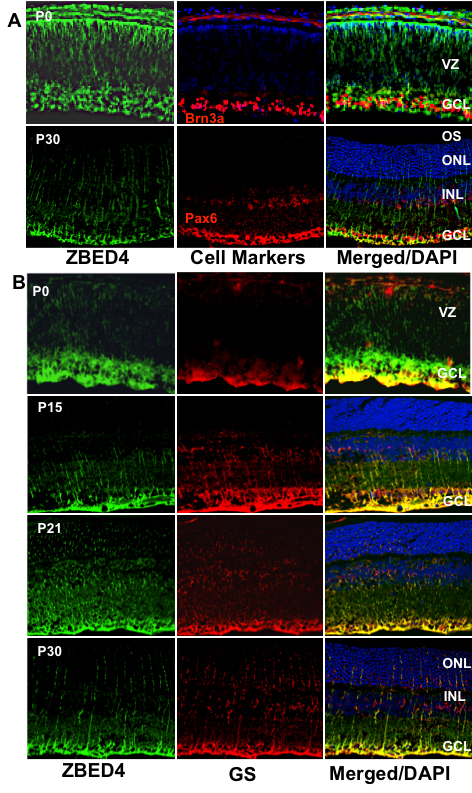
Co-localization of Zbed4 with cell markers in the developing mouse retina. Mouse retinal sections at several developmental stages were double labeled with antibodies against Zbed4 (green) and different cell markers: **A**: Upper panel: mouse Brn3a antibody was used to label nuclei of ganglion cells (red). Lower panel: mouse Pax6 antibody was used to label amacrine, ganglion and Müller cells (red). **B**: Mouse glutamine synthetase antibody was used to label Müller glial cells (red) at P0, P15, P21, and P30. Acronyms: VZ, ventricular zone; GCL, ganglion cell layer; OS, outer segments; ONL, outer nuclear layer; INL, inner nuclear layer. Appearance of yellow in the merged images indicates co-localization of ZBED4 with the different markers. Clearly, Zbed4 is not expressed in ganglion or amacrine cells of the retina, but is present in Müller cells throughout the life of the mouse.

To establish the association of Zbed4 in developing mouse retina with newly differentiating cell types, we performed a series of double-labeling experiments using antibodies to several specific cell markers. Figure 5A shows that at P0, a time at which ganglion cells are already placed at the innermost layer of the retina, there is no association of Zbed4 with these cells that are stained with the antibody against the transcription factor Brn3a, a ganglion cell-specific marker. In contrast, at P0, Zbed4 strongly co-localized with glutamine synthetase (Figure 5B), a Müller cell marker, in the endfeet of presumptive Müller cells. In fact, Zbed4 and glutamine synthetase were seen together throughout mouse retinal postnatal development (Figure 5B). Further, to rule out the co-localization of Zbed4 with amacrine cells, we double labeled the adult mouse retina (P30) with Zbed4 and Pax6 antibodies. No association of Zbed4 with amacrine cells was observed in the inner nuclear and ganglion cell layers (Figure 5A) of mouse retinal sections.

## Discussion

Despite the general similarities between human and mouse retinas, there are notable differences between the two species in the number, ratio, and distribution of photoreceptor cells and in their morphological characteristics and physiologic responses. The human retina has a larger proportion of cone photoreceptor cells than the mouse retina and it contains the macula, a specialized area that is not present in the mouse. In addition, mice are dichromats, whereas humans are trichromats. Therefore, spatial and temporal differences in gene expression patterns and protein function are expected between humans and mice.

In the present study we found that although the mouse *Zbed4* mRNA, which encodes a 1,168-amino acid protein, has the same structural features of its human ortholog [1], there are differences between human and mouse retinas related to the spatial expression of the ZBED4 and Zbed4 proteins.

It is well known that before birth in the mouse, during E16 to P0, ganglion cells are present in the innermost area of the retina (vitreal side), whereas the outer retina consists primarily of undifferentiated neuroblastic cells [10,11]. Since cone photoreceptors become postmitotic in the early embryonic period, these cells can be detected beneath the outer limiting membrane [10-12]. To determine which retinal cells expressed the Zbed4 protein, we performed several immunolocalization experiments at different times during mouse embryonic and postnatal development, using double labeling with rabbit polyclonal antibody against the mouse Zbed4 N-terminus and antibodies for specific cell markers. The Zbed4 antibody was purified and characterized in a similar manner to that for the human ZBED4 antibody [1] and it was shown to be Zbed4-specific. Our data showed that at P0, Zbed4 does not co-localize with Brn3a, a ganglion cell marker (Figure 5A). However, there is at this time strong staining of Zbed4 in the retinal innermost layer and some staining at the outermost layer as well as throughout the retina (Figure 5A). It is possible that at this stage of development cones express Zbed4, but we could not verify this because most of the available markers for cones localize to their outer segments; in the mouse, outer segments develop after several postnatal days. Therefore, at the stage in development between E16 and P0, we can only indicate that Zbed4 is present in neuroblasts of the mouse retina.

At P0 and later on during postnatal development, we found that Zbed4 co-localizes with glutamine synthetase, an established marker for glial Müller cells (Figure 5B). Zbed4 immunoreactivity decreases from that seen at early postnatal life at the choroidal border of the retina and is intense at the endfeet of Müller glial cells from P11 onward (Figure 3B). This can also be seen in Figure 4A,B, which show immunostaining localized both to endfeet and processes of Müller cells throughout the adult mouse and rat retinas. Thus, developmental patterns of spatial and temporal expression of Zbed4 suggest a possible role for this protein in retinal morphogenesis.

It may be speculated that the different cell distribution of Zbed4 in human and mouse retinas is related to the different structural features of the gene in these species. In addition, we have previously mentioned that there is only 82% homology between human and mouse Zbed4. Since zinc finger proteins of the BED class not only bind to DNA but also to RNA or proteins [13], contributing to many diverse cellular processes such as transcriptional regulation, mRNA stability and processing, and protein turnover, variations in amino acid sequences may lead to differences in localization or function of the corresponding proteins. This may explain why, while human ZBED4 showed more prominent nuclear and cytoplasmic expression in cone cells than in the endfeet of Müller cells [1], in adult mouse retina we observe strong expression of Zbed4 in the endfeet and processes of Müller cells but no immunostaining in cones. We should point out, however, that even when adult mouse cone photoreceptors may have minimal levels of Zbed4 protein (that we cannot detect), they have abundant *Zbed4* mRNA, as we have previously shown [1].

Interestingly, expression of Zbed4 in the nuclear fraction of mouse retina follows the opposite pattern to that in the cytoplasmic fraction during postnatal development (Figure 2), decreasing from early life, when retinoblasts have not yet differentiated, throughout the maturation process of all retinal cells that ends by adulthood. This reinforces the idea that Zbed4 may have an important role in retinal morphogenesis and development. Furthermore, we have recently discovered that Zbed4 binds in vivo to specific elements in the promoter region of retinal genes, positively regulating their transcriptional activity. In other words, Zbed4 behaves as a transcription factor (Mokhonov, Gribanova, Akhmedov, and Farber, data not shown). On the other hand, the presence of more Zbed4 in the cytoplasm of older than in younger retinal cells may indicate its possible involvement in other functions, such as modulation of other proteins activity by interaction with them. In fact, mouse Zbed4, like its human ortholog, has two nuclear receptor-interacting motifs (LXXLL) characteristic of co-activators/co-repressors of nuclear hormone receptors [13]. We have previously shown the presence of estrogen receptor α in nuclei of retinal cells [14], and it is possible that Zbed4 interacts with this nuclear receptor, resulting in transcriptional activation. The fact that Zbed4 is abundantly expressed in ovaries (Figure 1) also supports Zbed4 possible direct or indirect association with hormone receptors. In addition, hormone receptors have been demonstrated to play a critical role in retinal cell proliferation, differentiation, and development [15]. Interaction of Zbed4 with these receptors could be regulating these functions. Further studies, including the generation of knockout mice, may help to elucidate the physiologic functions of Zbed4 in the cellular processes that occur in the nuclei and cytoplasm of Müller cells.
